# PICS-Ord: unlimited coding of ambiguous regions by pairwise identity and cost scores ordination

**DOI:** 10.1186/1471-2105-12-10

**Published:** 2011-01-07

**Authors:** Robert Lücking, Brendan P Hodkinson, Alexandros Stamatakis, Reed A Cartwright

**Affiliations:** 1 Department of Botany, The Field Museum, 1400 South Lake Shore Drive, Chicago, IL 60605-2496, USA; 2 Department of Biology, Box 90338, Duke University, Durham, NC 27708-0338, USA; 3 The Exelixis Lab, Scientific Computing Group, Heidelberg Institute for Theoretical Studies, Schloss-Wolfsbrunnenweg 35, D-69118, Heidelberg, Germany; 4 Department of Biology and Biochemistry, University of Houston, 369 Science & Research Bldg 2, Houston, TX 77204-5001, USA

## Abstract

**Background:**

We present a novel method to encode ambiguously aligned regions in fixed multiple sequence alignments by 'Pairwise Identity and Cost Scores Ordination' (PICS-Ord). The method works via ordination of sequence identity or cost scores matrices by means of Principal Coordinates Analysis (PCoA). After identification of ambiguous regions, the method computes pairwise distances as sequence identities or cost scores, ordinates the resulting distance matrix by means of PCoA, and encodes the principal coordinates as ordered integers. Three biological and 100 simulated datasets were used to assess the performance of the new method.

**Results:**

Including ambiguous regions coded by means of PICS-Ord increased topological accuracy, resolution, and bootstrap support in real biological and simulated datasets compared to the alternative of excluding such regions from the analysis a priori. In terms of accuracy, PICS-Ord performs equal to or better than previously available methods of ambiguous region coding (e.g., INAASE), with the advantage of a practically unlimited alignment size and increased analytical speed and the possibility of PICS-Ord scores to be analyzed together with DNA data in a partitioned maximum likelihood model.

**Conclusions:**

Advantages of PICS-Ord over step matrix-based ambiguous region coding with INAASE include a practically unlimited number of OTUs and seamless integration of PICS-Ord codes into phylogenetic datasets, as well as the increased speed of phylogenetic analysis. Contrary to word- and frequency-based methods, PICS-Ord maintains the advantage of pairwise sequence alignment to derive distances, and the method is flexible with respect to the calculation of distance scores. In addition to distance and maximum parsimony, PICS-Ord codes can be analyzed in a Bayesian or maximum likelihood framework. RAxML (version 7.2.6 or higher that was developed for this study) allows up to 32-state ordered or unordered characters. A GTR, MK, or ORDERED model can be applied to analyse the PICS-Ord codes partition, with GTR performing slightly better than MK and ORDERED.

**Availability:**

An implementation of the PICS-Ord algorithm is available from http://scit.us/projects/ngila/wiki/PICS-Ord. It requires both the statistical software, R http://www.r-project.org and the alignment software Ngila http://scit.us/projects/ngila.

## Background

Sequence alignment is the most critical step in molecular phylogenetic analysis. It defines homologous sites and putative evolution of site-specific variation [1-5]. However, sequence portions in multiple sequence alignments (MSA) may have low alignment confidence, that is they are ambiguously aligned (often called 'ambiguous regions') due to a variable number of indels, and thus different alignment solutions with identical cost scores are possible [6,7]. Such portions are usually excluded from further analysis [8-10], as methodologies often only work on a single MSA, and ambiguities require subjective prioritization of a single alignment solution. Length-variable regions also have an increased probability of homoplastic evolution. However, it is recognized that ambiguously aligned portions do carry substantial phylogenetic signal [11-13].

Methods that do not require a single MSA provide one solution to this problem. Direct optimization (DO) optimizes alignments and trees simultaneously under parsimony, likelihood, or in a Bayesian framework [14-21]. However, while DO can handle uncertainty in alignments due to sequence length variation, it is computationally intensive and support is usually calculated by sampling of alternative alignments and trees, as credible intervals, posterior probabilities, or Bremer support. These are not directly comparable to bootstrap support, which in DO is only possible by creating pseudo-alignments, which defies the purpose of DO. Furthermore, it is also disputed whether alignments with optimized cost or likelihood scores under a given setting will return optimized tree topologies [22-26].

An alternative to DO or to excluding ambiguous regions is the separate analysis of indels and encoding them as non-DNA characters [13]. The simplest approach is to encode residues by sequence length (each length receiving a different code) or coding them as identical or different without measuring the degree of identity or difference [27]. Tools such as SNAP [28,29] and MAFFT [30-32] include options to encode short indels (gaps). More complex methods dealing with larger gaps include frequency-based, motif-based, and pairwise distance-based methods. Frequency-based methods such as 'ambiguous regions coding', ARC [33] calculate the relative frequencies of bases and base pairs and their spatial distribution within sequences of ambiguously aligned regions. Motif- or 'word'-based methods search for common 'substrings' in ambiguously aligned sequences [34]. Applications include ARC and 'N-local decoding' [35,36]. The only pairwise distance-based method appears to be 'integration of ambiguously aligned sequences', INAASE [37], which encodes each region as a single character with a corresponding step matrix. INAASE recovers phylogenetic signal contained in ambiguous regions rather accurately, whereas ARC produces topologies that do not always agree with the underlying signal [38]. The size of step matrices is limited to a fixed number of states: 32 states in PAUP* 4.0b10 (as an unsigned integer has 32 bits), 64 states on newer CPUs [39], therefore INAASE cannot be applied to alignments that include a large number of taxa or complex and highly variable ambiguous regions. In addition, the search time (including bootstrapping) is increased about 10 to 100 times depending on the number and size of the step matrices included (unpubl. data based on comparative analysis of datasets on single-processor APPLE Aluminium G4 Powerbook and DELL Inspiron 1720 laptop computers).

The solution to the size and performance limitations of step-matrix-based analysis is to transform the multidimensional step matrix into unidimensional scores prior to phylogenetic analysis. This way, computing pairwise alignment scores can be applied to a theoretically unlimited number of OTUs and to ambiguous regions with high length variation and complexity. This is achieved by ordinating the step matrix and dissecting it into perpendicular axes. The axis coordinates for each OTU can then be used to obtain codes to replace the ambiguously aligned regions. The ordination method of choice must accept similarity (identity) or dissimilarity (cost) matrices as input, which excludes principal component analysis (PCA).

Three commonly used methods can ordinate OTUs based on identity or distance matrices: polar ordination (Bray-Curtis), non-metric multidimensional scaling (NMS), and principal coordinates analysis or 'metric multidimensional scaling', PCoA [40,41]. NMS has become the default ordination method for ecological data [41-43], but the ordination is computed via an iterative numerical procedure that starts from a random starting configuration. Therefore, sample points that have identical original data scores (e.g., identical sequences), and hence should fall on exactly the same point in the ordination diagram, will instead slightly disperse. This would affect sequence ordination, since identical sequences would result in slightly different ambiguous region codes. Polar ordination and PCoA do not exhibit this problem, and the latter method has the advantage of being an eigenvector analysis, which calculates the degree of variance explained by each axis [40,41,44]. PCoA has been applied to sequence ordination, although mostly for visualizing sequence and tree spaces [13,45]; http://pbil.univ-lyon1.fr/mva/pco.php). Because of its properties, it is the default method for the algorithm proposed here.

In this paper, we describe the computational procedure to encode ambiguous regions: (1) compute pair-wise distance matrices for ambiguous regions of an alignment, (2) ordinate the distance matrices, and (3) encode the ordination scores and integrate them into a phylogenetic data matrix. Our novel method, PICS-Ord, was tested using three biological and 100 simulated datasets. One biological dataset (100 OTUs, mtSSU) was extracted from a large dataset of over 600 OTUs and three genes (mtSSU, nuLSU, RPB2) of the lichenized fungal family Graphidaceae [[46,47]; unpubl. data], whereas the second dataset represented 706 OTUs and one partial gene (ITS) of the family Physciaceae. The third dataset, representing 1814 OTUs and one partial gene (ITS) of the lichen family Parmeliaceae, was used to assess computational speed for large datasets. Both similarity matrices created by ClustalW [48] and Ngila [49] were tested.

## Results

### Maximum Parsimony

**Figure 1:**
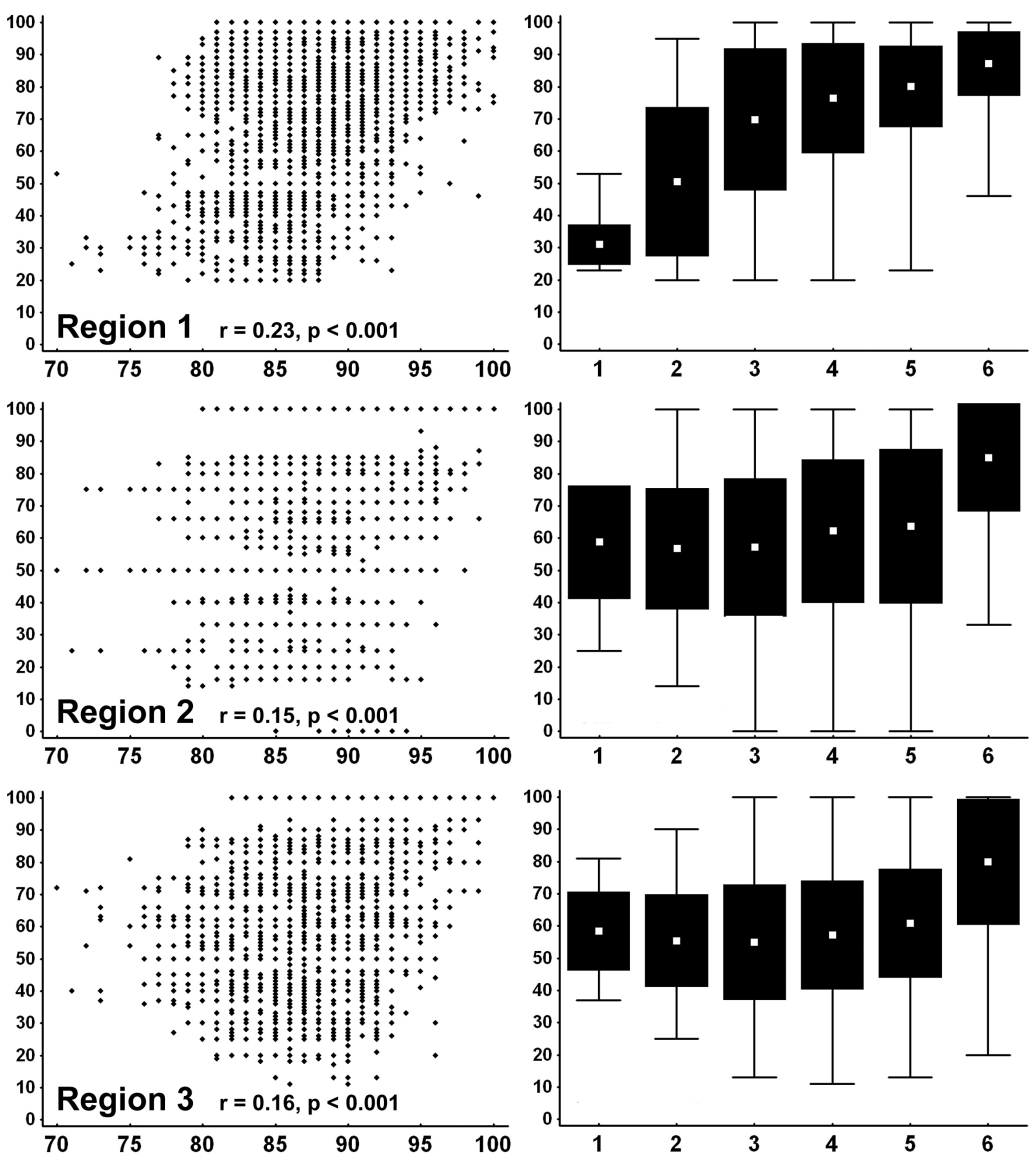
**Correlation between Clustal sequence identity scores of the non-ambiguous alignment portion (x-axis) and each of the ambiguous regions (y-axis)**. Left column: scatterplots of sequence identity scores, with linear correlation tested using Spearman rank correlation. Right column: same data but categorized to show emerging pattern (1: 70-75%; 2: >75-80%; 3: >80-85%; 4: >85-90%; 5: >90-95%; 6: >95-100%). Box plots indicate mean, standard deviation, and maximum/minimum values.

The three ambiguous regions of the 100-OTU Graphidaceae dataset showed different degrees of congruence with the non-ambiguous alignment portion (Figure 1). The phylogenetic signal of region 1 correlated better with that of the non-ambiguous alignment portion compared to regions 2 and 3. For regions 2 and 3, identity scores between ambiguous sequences were on average higher than expected when identity scores between non-ambiguous alignment portions were low, suggesting some degree of homoplasy through a saturation effect in short ambiguous sequences.

**Figure 2:**
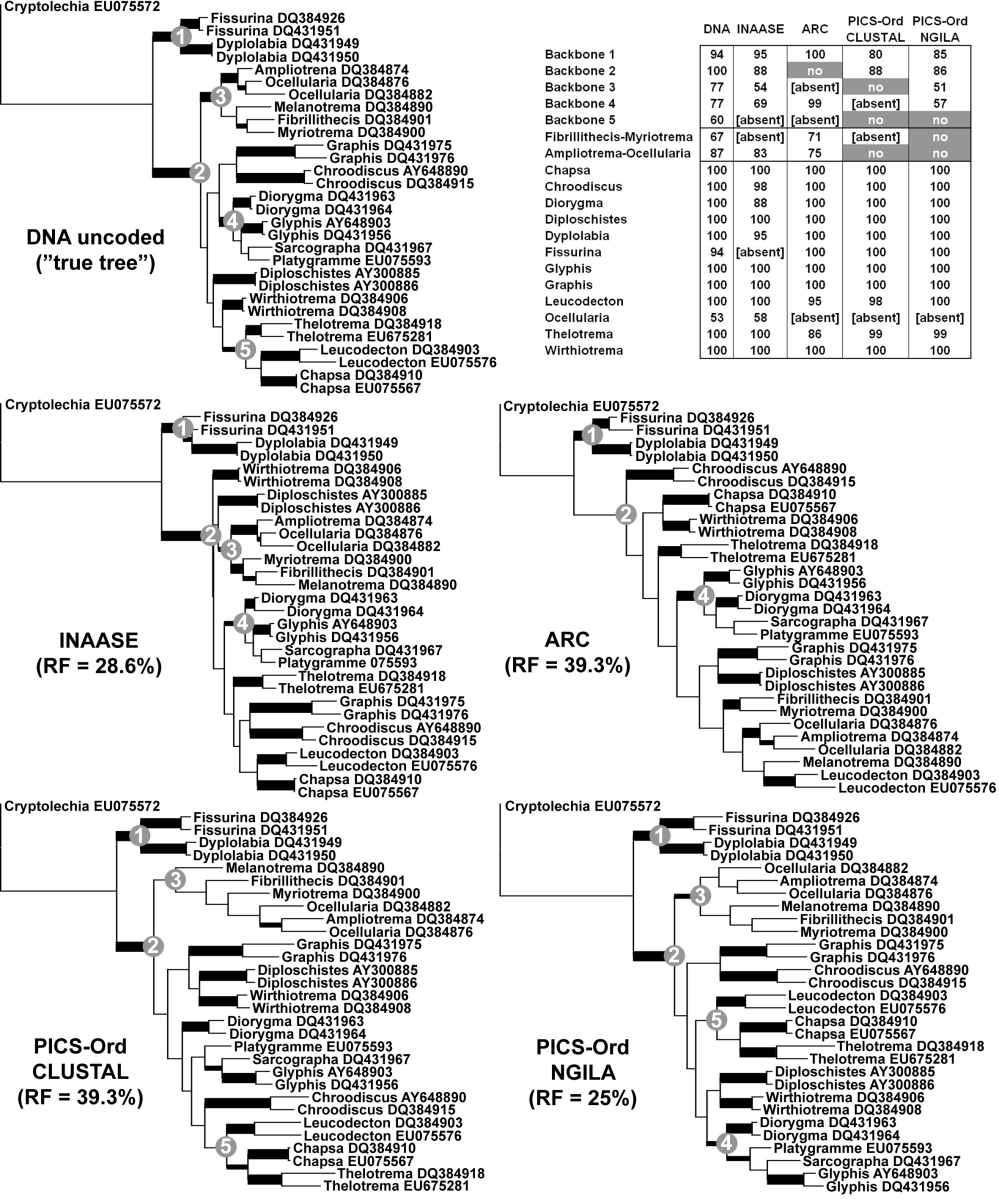
**Maximum parsimony trees computed from the non-ambiguous alignment portion, using original DNA data and data recoding by means of INAASE, ARC, PICS-Ord with Clustal sequence identity and PICS-Ord with Ngila zeta cost scores**. The five major backbone nodes that are also supported in multigene studies are indicated by grey circles. Branches with good or strong support (70% or higher) and indicated by thick lines and branches with weak support (less than 70%) by slightly thickened lines. Exact bootstrap support values for backbone and terminal nodes are indicated in the table in the upper right corner.

Recoding of the non-ambiguous alignment portion of 31 OTUs with ARC, INAASE, and PICS-Ord with CLUSTAL, and PICS-Ord with Ngila distances, resulted in partially deviating maximum parsimony topologies ('distortions') when compared to the tree derived from the uncoded, original DNA alignment (Figure 2). ARC and PICS-Ord with CLUSTAL distances resulted in comparatively high relative RF (Robinson Foulds) distance values of nearly 40%, whereas INAASE and PICS-Ord with Ngila distance gave better values near 25%. INAASE recoding produced two conflicts (conflictive topology with bootstrap support 70% or higher) compared to the uncoded DNA topology: *Fissurina *was resolved as paraphyletic and the *Graphis*-*Chroodiscus *clade was nested within the *Chapsa*-*Leucodecton*-*Thelotrema *clade. ARC recoding also exhibited two conflicts: non-monophyly of the *Chapsa*-*Leucodecton*-*Thelotrema *clade and *Leucodecton *being nested within the *Ampliotrema*-*Fibrillithecis*-*Melanotrema*-*Myriotrema*-*Ocellularia *clade. PICS-Ord recoding with Clustal distances resulted in one conflict, viz. paraphyly of the *Diorygma*-*Glyphis*-*Platygramme*-*Sarcographa *clade, whereas. PICS-Ord recoding with Ngila distances did not show any conflict with the topology derived from the uncoded DNA.

All recoding methods resulted in some loss of backbone support, whereas support for terminal nodes remained largely unchanged (Figure 2). ARC did not support the large sister clade of the basal split, which received absolute support (100%) in uncoded DNA analysis and good support (86-88%) with the other recoding methods. Genus group support was generally lower with encoded data and especially under ARC and PICS-Ord. Genus support was high for all recoding methods and especially using PICS-Ord with Ngila distances, with the exception of INAASE which recovered *Diorygma *with lower support and did not recover the otherwise strongly supported *Fissurina*. The best overall recoding performance (least amount of distortion) was thus found with PICS-Ord using Ngila distances. The performance of ARC was fairly poor, with high relative RF value, conflictive topology, and absence of support for the largest backbone node.

### Maximum Likelihood

**Figure 3:**
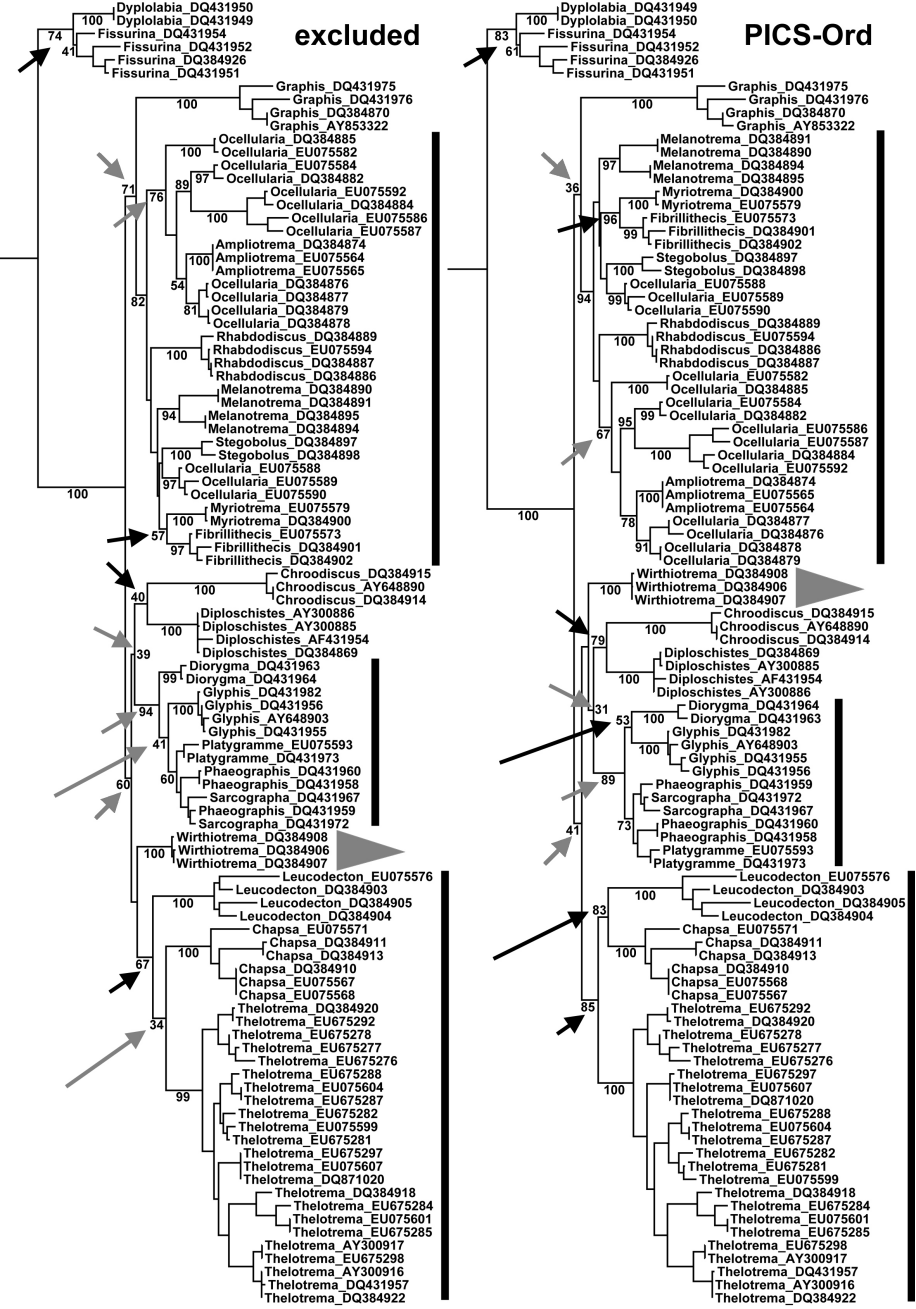
**Maximum likelihood trees computed from the 100-OTU Graphidaceae dataset with ambiguous regions excluded (left) and recoded using PICS-Ord with Ngila zeta cost scores (right)**. A GTR-Gamma model was applied to the DNA partition and a GTR model for the PICS-Ord code partition (GTR-CAT for rapid bootstrapping in both cases). Bootstrap support values are indicated next to the branches. Grey triangles indicate major clades with different position in both analyses, and black lines indicate clades with internal topology differing between analyses. Short arrows indicate nodes with increased (black) or decreased (grey) support under PICS-Ord and long arrows indicate nodes present either with ambiguous regions excluded (grey) or under PICS-Ord (black).

Maximum likelihood analysis of the 100-OTU Graphidaceae dataset with ambiguous regions either excluded or encoded using PICS-Ord (Ngila with zeta model) resulted in largely congruent topologies, with only one major clade switching positions between analyses (Figure 3). Three further major clades had internal topologies changed between analyses: the *Ampliotrema*-*Fibrillithecis*-*Melanotrema*-*Myriotrema*-*Ocellularia *clade, the *Diorygma*-*Glyphis*-*Phaeographis*-*Platygramme*-*Sarcographa *clade, and the *Chapsa*-*Leucodecton*-*Thelotrema *clade. However, this topological conflict was not supported, except for the clustering of *Chapsa *and *Leucodecton *under PICS-Ord. The two analyses involved 36 backbone and terminal nodes of interest. Of these, 14 nodes had absolute support (100%) in both cases (Figure 3). Fourteen further nodes had increased support under PICS-Ord, with an average increase of 23% for five backbone and genus group nodes and 6% for nine terminal genus nodes. Especially notable was the increase under PICS-Ord from 40% to 79% for the *Chroodiscus*-*Diploschistes *node and from 57% to 96% for the *Fibrillithecis*-*Myriotrema *node, two nodes that are supported in multigene studies (Rivas Plata et al., in prep.). For two genus group nodes (*Ampliotrema*-*Ocellularia *and *Phaeographis*-*Platygramme*-*Sarcographa*), support decreased slightly under PICS-Ord (average of 7%), whereas the two major backbone nodes of the large sister clade to *Fissurina*-*Dyplolabia *showed substantial decrease in support under PICS-Ord (71% to 36% and 60% to 41%, respectively). The two latter nodes are not recovered in multigene studies. The remaining four nodes appeared in one of the two analyses only: with ambiguous regions excluded, *Glyphis *clustered with the *Phaeographis*-*Platygramme*-*Sarcographa *clade and *Leucodecton *with *Thelotrema*, in both cases lacking support (41% and 34%, respectively), whereas under PICS-Ord, *Glyphis *clustered with *Diorygma *(53% support) and *Leucodecton *with *Chapsa *(83% support); the latter topologies are congruent with multigene studies. Inclusion of ambiguous regions under PICS-Ord thus did not only result in overall increased bootstrap support, but also in topologies that are more in accordance with multigene studies using mtSSU, nuLSU, and *RPB2 *[[46,47]; unpubl. data].

### Simulations

**Figure 4:**
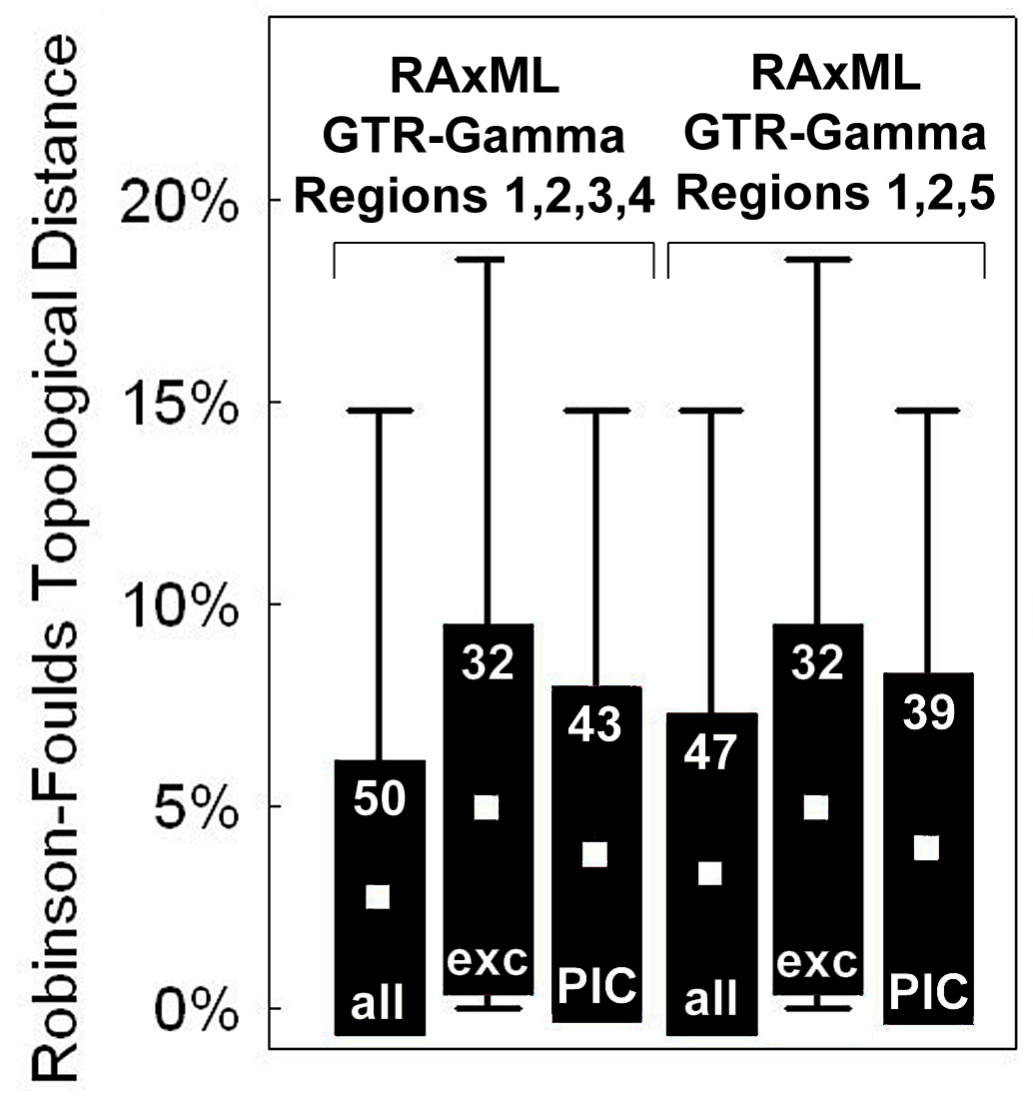
**Distribution of RF values of recovered tree topologies under different methodological approaches of excluding and including ambiguous sections in the simulated datasets (compared to the true tree from which the simulated datasets were generated); all = all sections pre-aligned, exc = ambiguous sections excluded, PIC = PICS-Ord coding (Ngila zeta model)**. Numbers in upper part of boxes indicate recovered true trees (out of 100). Box plots indicate mean, standard deviation, and maximum/minimum values.

We generated 100 simulated datasets of aligned sequences, each split into five partitions. Partitions 1 and 2 had unambiguous alignments, while 3-5 had different degrees of alignment ambiguity. Sections 1-4 were combined in one analysis, while 1, 2, and 5 in another. RAxML analysis of the 100 simulated datasets recovered the best trees when sections 1-4 (1+2+5; results below given in parentheses for each treatment) were trea-ted as pre-aligned without changes, with a mean relative RF value of 2.74% (3.33%) and recovering the true tree 50 (47) times out of 100 (Figure 4). Excluding ambiguous sections 3-5 resulted in less accurate topologies on average (relative RF = 4.93%): 56 (63) datasets gave identical trees compared to pre-aligned sections 1-4 (1-2, 5), seven (14) datasets resulted in improved topology, but 37 (23) datasets showed worse topology. Recoding ambiguous sections using PICS-Ord on average improved topologies under all three employed substitution models and particularly under GTR (RF = 3.81% and 3.96%, respectively): 59 (59) datasets gave identical trees compared to ambiguous sections excluded for sections 3-4 (5) encoded, 29 (27) datasets resulted in improved topology, and 12 (14) datasets gave a worse topology. Thus, recoding ambiguous sections under PICS-Ord resulted in identical or improved topologies compared to excluding ambiguous sections in 88 (86) out of 100 cases. All differences were statistically significant using a Wilcoxon matched pairs test (Table 1).

**Table 1:** Wilcoxon matched pairs test comparing the RF values of simulated datasets.

	1-2, 3-4 pre-aligned	3-4 excluded
3-4 excluded	*** (-)	NA
3-4 PICS-Ord	** (-)	** (+)
	**1-2, 5 pre-aligned**	**5 excluded**
5 excluded	*** (-)	NA
5 PICS-Ord	-- (-)	* (+)

Simulated datasets are given as sections 1-2, 3-4 or sections 1-2, 5 under different treatments of ambiguous regions (all included and pre-aligned or sections 3-4 and 5 excluded or PICS-Ord-recoded). ***/**/* = significant at the 0.001/0.01/0.05 level; -- = not significant; (+)/(-) = topology improved/worse.

**Figure 5:**
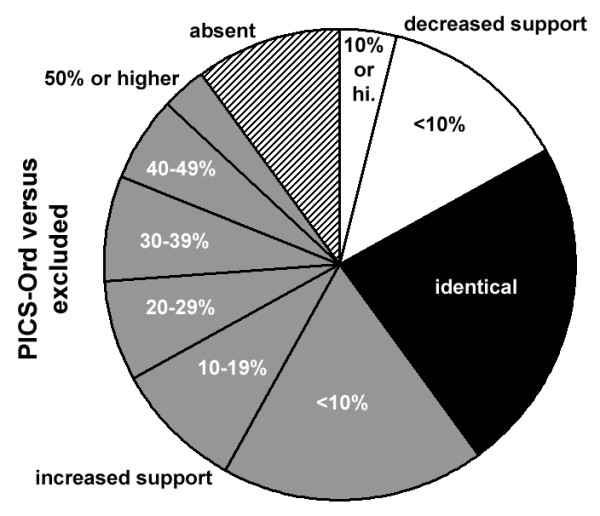
**Proportion of increased or decreased support values for 100 backbone, genus group, genus, species group, and species nodes of the 705-OTU Physciaceae dataset analysed under maximum likelihood with ambiguous regions either excluded or recoded with PICS-Ord**. Nodes were divided according to whether PICS-Ord recoding performed better than, identical to, or worse than excluding ambiguous regions. Numbers in parentheses indicate mean difference in support values using PICS-Ord versus the other two methods.

The 705-OTU Physciaceae dataset showed 100 nodes at the backbone, genus group, genus, species group, and species level (with at least three samples per species; tree not shown). Eighteen nodes were present under PICS-Ord but absent when ambiguous regions were excluded; of these, nine had support values ranging between 14% and 69% and nine had values ranging between 77% and 100% under PICS-Ord (Figure 5). A total of 36 nodes had higher support under PICS-Ord compared to both alternative treatments, and the differences ranged between 21.5% and 54% for 15 nodes, between 11% and 19.5% for ten nodes, and between 2% and 10% for 11 nodes. For another ten nodes, PICS-Ord gave higher support values compared to one of the alternative methods and identical values compared to the other, with an average increase of 4.4%. Nineteen nodes behaved identically for all three methods, with support values of 100%. For seven nodes, PICS-Ord gave higher support values compared to one alternative method (average increase 8.9%) but lower values compared to the other (average decrease 4.2%). The remaining ten nodes showed lower support values for PICS-Ord compared to both alternative methods, with an average decrease of 6.0% (Figure 5). PICS-Ord thus showed overall increased support for 64 nodes and overall decreased support for only ten nodes, with an average increase over all nodes of 10.3% and a maximum increase for one node versus ambiguous regions excluded of 78%. All nodes with increased support under PICS-Ord or which appeared only under PICS-Ord correspond to clades and taxa that are supported in two-gene or multigene studies including also mtSSU and nuLSU [[46,47]; unpubl. data].

## Discussion

Our study shows that ordination of distance matrices, while introducing a small amount of distortion, recovers phylogenetic signal remarkably well. For non-ambiguous data with a 'known' topology derived from uncoded DNA, INAASE and PICS-Ord with Clustal identity scores performed similarly, with most but not all clades recovered accurately. PICS-Ord with Ngila zeta cost scores slightly outperformed both methods, whereas the performance of ARC could be best characterized as fair. Problems with ARC have been reported [38] and are based on the fact that the recoding method used in ARC is not distance-based but encodes sequences based on length and relative frequency of individual bases and base pairing patterns [33]. Under certain, usually rare circumstances, this can lead to erroneous codes, as the following example illustrates: consider sequences (1) TTGGCCAACCGGTT, (2) AACCGGTTGGCCAA, and (3) AGCCAGCTGGCTAA. Sequences (2) and (3) are more similar to one other, differing in four transitions only, whereas sequences (1) and (2) are dissimilar. However, because they have similar base and base pair frequencies, ARC will encode sequences (1) and (2) as being more similar to one other and sequences (2) and (3) as being dissimilar, as the ARC codes demonstrate: (1) 00000000000000000000000, (2) 01001100000000100001001, and (3) 01001011111111211112112. Therefore, ARC may not only recover topologies in conflict with non-ambiguous portions of the alignment but also in conflict with the phylogenetic signal contained in the ambiguous regions. Distance-based methods avoid this problem. INAASE has been shown to perform well when the dataset is sufficiently small, recovering phylogenetic signal with great accuracy, even though the actual number of codes is very small, with a single character representing each ambiguous region [37,38]. For large datasets with over 32 distinct sequence patterns in ambiguous regions, PICS-Ord with Ngila zeta cost scores offers a good and fast alternative. Zeta cost scores slightly outperformed simple identity and cost scores in our analysis, confirming the results of previous studies [25,49].

Since PCoA ordination is an eigenvector analysis, the eigenvalues can be used to assess the amount of information represented by each ordination axis and be implemented as weight factor. However, if the PICS-Ord codes are used as ordered characters, the coding method encodes the ordination scores proportionally to the amount of variance explained by each axis, and a weighting factor will not markedly affect the overall performance. Weighting of the axes based on eigenvalues is recommended when the codes (equivalent to columns or sites) produced by PICS-Ord are analyzed as unordered characters or in a GTR model under maximum likelihood, although tests (results not shown) did not suggest marked changes in topology or support with unweighted or weighted PICS-Ord codes. One might also consider weighting to balance the influence of DNA versus PICS-Ord characters in a partitioned dataset. However, in general this will not be necessary. The number of code columns (sites) retained by PICS-Ord for each ambiguous region depends on the number of different sequence motifs present, with a maximum number corresponding to the number of OTUs. In our experience, only about 25-35% of sites will have positive eigenvalues and about 15-25% will be retained after removing invariant sites. The first ambiguous region each of the 100-OTU Graphidaceae, the 706-OTU Physciaceae, and the 1814-OTU Parmeliaceae dataset retained 20, 172, and 320 sites, respectively. In addition, only the first few axes will be clade-informative, that is they contain structure largely congruent with clades resolved by non-coded DNA, and hence increase clade support, whereas the higher axes tend to be 'near-constant'. In a typical dataset of 100-1000 OTUs, the number of sites retained by PICS-Ord for each ambiguous region that are 'clade-informative', will be roughly 5-25. In ITS datasets containing roughly 450 unambiguously-aligned nucleotide sites, the 'clade-informative' PICS-Ord axes, assuming 2-3 ambiguous regions, would therefore add roughly about 15-75 sites, replacing originally ambiguous portions of roughly 100-150 bases in length.

The usefulness of including ambiguous regions in phylogenetic analyses and the performance of the corresponding recoding method can be evaluated using two criteria: improved confidence (statistical support) and improved topology (phylogenetic accuracy). Topology can be judged indirectly: when two different methods applied to the same dataset result in topological differences, but under certain conditions the topologies converge, this can be seen as improvement towards phylogenetic accuracy, as long as the resolution does not decrease and no novel topologies appear [50,51]. Ambiguous regions likely contain homoplastic phylogenetic signal which could mask the signal contained in non-ambiguous portions of the alignment. A simple way to test this is to plot distance matrices obtained from non-ambiguous and ambiguous regions against each other. If there is an acceptable level of congruence, one would expect that inclusion of ambiguous regions by means of a coding method should improve support and/or topology. The best way of testing these criteria is through the use of simulation studies [52,53]. However, simulated data are typically not as 'messy' as real biological data, and only a combined approach inclu-ding biological and simulated data allowed us to assess the performance of our novel recoding method.

The simulation study showed that excluding ambiguous regions resulted in significantly worse topologies and that including them by means of PICS-Ord allowed the recovery of a substantial part of the phylogenetic signal contained therein. The most accurate topologies were obtained when analyzing the simulated datasets unchanged ('as is'); however, since in real biological data we cannot know the true alignment, the inclusion of ambiguous regions by means of recoding, rather than excluding them, is the next best option. In PICS-Ord, recoding ambiguous regions is based on a single optimal solution for each pairwise alignment given NGILA's model of log-affine gap costs, and the transformation of these pairwise alignments into distances reduces the risk of misinterpretation of positional homologies compared to frequency-based methods such as ARC.

The potential power of recovering phylogenetic signal contained in ambiguous regions is shown in our analysis of the 100-OTU Graphidaceae dataset. The topology and support obtained when including ambiguous regions of the mtSSU gene by means of PICS-Ord matches the topology and support obtained by a three-gene tree [unpubl. data] better than the topology based on exclusion of ambiguous regions. Published 2-gene and 3-gene phylogenies of Graphidaceae [[46,47]; unpubl. data] recovered the *Fissurina*, *Ocellularia*, *Phaeographis*, and *Thelotrema *clades with strong support (90-100% maximum likelihood bootstrap and 0.98-1.00 posterior probability). In our approach, PICS-Ord recoding for these clades increased support by 9% for the *Fissurina *clade and by 18-20% for the *Ocellularia *and *Thelotrema *clades. *Graphis *was supported sister to the *Ocellularia *clade when ambiguous regions were excluded but that support disappeared when using PICS-Ord recoding; in 2-gene and 3-gene phylogenies, *Graphis *does not appear as sister to the *Ocellularia *clade. Similarly, *Wirthiotrema *appeared sister to the *Thelotrema *clade when ambiguous regions are excluded, but sister to a clade including *Diploschistes *under PICS-Ord, which is more in line with published 2-gene and 3-gene phylogenies. This indicates that the phylogenetic signal contained in the ambiguous portion of the mtSSU gene is congruent with the phylogenetic signal contained in other genes (nuLSU, *RPB2*) and therefore should not be excluded from phylogenetic analysis. The predictive power contained in ambiguous portions of the mitochondrial small subunit ribosomal DNA and in the nuclear internal transcribed spacer ribosomal DNA, especially at lower hierarchical level, make these genes promising candidates for DNA barcoding in Ascomycota [54-56].

During our study, we made some preliminary comparisons (results not shown) between PICS-Ord and direct optimization methods such as POY, BALi-Phy, PRANK, and SATè [14-20,57]. Although PICS-Ord recoding introduces a slight distortion of the original data, we did not find that topological accuracy was increased when using DO instead of PICS-Ord recoding, either with the simulated or with the biological datasets. In fact, some of the DO methods tested consistently returned less accurate topologies, even if the alignment and tree scores were improved under variable cost settings. In addition, inference time was substantially increased for all DO methods compared to analyzing mixed datasets including PICS-Ord codes under maximum likelihood in RAxML, by a factor of ten to one hundred or even more depending on the method. The detailed results of our comparison with DO methods will be presented in a forthcoming publication.

PICS-Ord thus offers a simple and cheap-to-compute alternative to direct optimization and recoding methods such as INAASE [37], when phylogenetic trees are derived from fixed multiple alignments with substantial ambiguous portions. For smaller datasets capable of being handled by INAASE (maximum of 32 or 64 sequence patterns per ambiguous region), INAASE and PICS-Ord coding with Clustal give fairly similar results, and INAASE might be the preferred method, since the step matrices reflect the actual sequence distances before ordination. However, INAASE can only be implemented with parsimony analysis and not within a Bayesian or maximum likelihood framework. Also, PICS-Ord with Ngila zeta costs scores outperformed INAASE, and application of a power-law model of indel evolution was shown to be superior to other methods for sequences with variable indels [25,49]. A criticism of PICS-Ord might be that the resulting codes are abstract entities and do not directly correspond to DNA or phenotype data. However, since the ordination axes are perpendicular to each other and the ordination space is a reflection of the original pairwise distance matrix space, the PICS-Ord codes can be interpreted as mathematically independent components of the original sequences' distances, thus fulfilling two important requirements for their phylogenetic analysis: mathematical independence and reflecting the original distance space. Besides easy computation of ambiguous region codes and seamless integration of data partitions (no user-defined step matrices are required), PICS-Ord allows for a practically unlimited number of OTUs and sequence patterns to be analyzed.

The modularity of PICS-Ord allows for flexible parameter settings, including transition:transversion ratio and gap penalties similar to those of INAASE when calculating simple pairwise cost scores in Ngila [25,49]. Alternative distance measures other than those provided by Clustal (identity) or Ngila (simple or zeta cost scores) are also conceivable, such as those based on 'N-local decoding' [35,36]. Another possibility for fine-tuning PICS-Ord lies in the number of ordination axes selected for recoding and in the way the principal coordinates are encoded. This allows for adjustments of PICS-Ord codes with respect to the relative length of ambiguous regions within a given alignment. The fact that PICS-Ord codes are simple integer values permits combined analysis of DNA and PICS-Ord code partitions in a Bayesian framework [58], with up to 6-state ordered codes, and under maximum likelihood with RAxML 7.2.6, using a GTR or MK model or characters as ORDERED, with up to 32 states. This was previously impossible with mixed letter/integer codes or codes representing user-defined step matrices. In addition to the improved topological accuracy, a further argument for using PICS-Ord versus direct optimization or INAASE is computational speed: recoding the ambiguous region in our 705-OTU dataset on a dual-core INTEL processor took two minutes, and analysis of the partitioned dataset under maximum likelihood in RAxML on the same machine required about 36 hours including rapid bootstrapping (100 replicates). For the 1814-OTU Parmeliaceae dataset, recoding took about 35 minutes for each region and maximum likelihood analysis including rapid bootstrapping (100 replicates) in RAxML lasted eight days. This is comparable to the time a 50-100-OTU dataset would have taken to be analysed on the same processor under maximum parsimony in PAUP with ambiguous regions included as INAASE step matrices. Computational speed can further be substantially increased when running the software on multi-processor computers and web servers [59]. Since PICS-Ord uses a set of integer codes to represent each ambiguous region for a given OTU, another problem of INAASE is avoided: the limited number of available symbols when coding an entire ambiguous region as a single character.

While PICS-Ord recoding was here applied to DNA data, the underlying method can be used to incorporate any kind of multidimensional distance matrix as unidimensional columns in a phylogenetic dataset and hence simplify the analytical approach and considerably increase computational speed.

## Conclusions

PICS-Ord offers a simple and fast method to recode regions in multiple sequence alignments that exhibit low alignment confidence scores ('ambiguous regions') and include them as separate partition in phylogenetic analyses. PICS-Ord can deal with datasets of practically unlimited size and the codes can be analyzed under maximum likelihood and Bayesian approaches, thus eliminating the disadvantages of previously available methods of ambiguous region coding while retaining the relative accuracy of distance-based recoding methods. The incorporation of Ngila allows for a variety of models of indel evolution to be implemented in the coding process, including a power-law zeta model. PICS-Ord is especially useful for phylogenetic analyses that use ribosomal genes (mitochondrial small subunit, mtSSU; nuclear internal transcribed spacer, ITS), as these genes are difficult to align even across closely related taxa, and is therefore a useful alternative to computationally intensive methods that optimize alignments and trees simultaneously. For typical mtSSU and ITS datasets or other multiple sequence or protein alignments that contain portions aligned with low confidence but containing phylogenetic signal, PICS-Ord coding will substantially improve topology and increase support compared to excluding such portions from the analysis.

## Methods

### Biological and simulated datasets and delimitation of ambiguous regions

Three datasets of real biological data were analyzed. One dataset was extracted from a larger dataset of the lichen family Graphidaceae that originally consisted of three genes (nuLSU, mtSSU, *RPB2*) and 280 morphological characters for over 600 OTUs [[47]; unpubl. data]. The extracted sample dataset comprised 100 OTUs representing eight major clades and 19 subclades. Only the mitochondrial small subunit ribosomal DNA (mtSSU) was used for this study, as it exhibited substantial ambiguity. The mtSSU alignment had a total length of 963 positions, with 738 sites corresponding to non-ambiguous portions. The second and third dataset comprised 705 and 1814 OTUs of the lichen families Physciaceae and Parmeliaceae, respectively, both representing the internal transcribed spacer (ITS) of the nuclear ribosomal DNA. The Physciaceae alignment had a total length of 564 sites, with 446 corresponding to non-ambiguous portions, whereas the Parmeliaceae alignment had 634 sites, with 456 corresponding to non-ambiguous portions.

The delimitation of ambiguous regions is in itself a difficult task [13,37]. Several methods have been proposed, such as 'culling' and 'elision' [60,61]. However, these methods are very conservative and usually identify portions of non-ambiguous alignments as ambiguous. Recent approaches include HoT (Heads or Tails), which uses an approach of comparing sequences aligned in original or reversed order [6,62,63], and the GUIDANCE scores [7,64]. Here, we used GUIDANCE through the web server at http://guidance.tau.ac.il, plus a manual approach to assess alignment confidence scores and delimit ambiguous regions.

After initial multiple alignment using ClustalW2 [48,65] and MAFFT [30-32], we identified 5-base-long conserved flanks of highly length-variable ambiguous regions using the likelihood of a given n-sized base combination to have evolved by chance; a conserved 5-base-long motif was determined if the maximum pairwise distance across all OTUs was 2.0 (with a cost of 0.5 for transitions and 1 for transversions). The GUIDANCE scores and the 'manual' method returned remarkably similar results: for the 100-OTU Graphidaceae dataset, GUIDANCE delimited regions with low alignment confidence as positions 29-126, 513-542, and 698-807 in the GUIDANCE-MAFFT alignment, whereas our manual delimitation, which was used in subsequent analyses, resulted in positions 28-127, 514-545, and 699-809. The three ambiguous regions recognized for that dataset showed length variation of 30 to 94 bases (region 1), 4 to 31 bases (region 2), and 6 to 100 bases (region 3), respectively. For the 705-OTU Physciaceae dataset, two ambiguous regions were identified, with length variation of 9-73 (region 1) and 22-45 (region 2), respectively. The 1814-OTU Parmeliaceae alignment had three ambiguous regions, with length variation of 52-72 (region 1), 27-46 (region 2), and 19-33 (region 3)

In addition to the three biological datasets, we generated 100 simulated datasets using DAWG 1.2 [52]. Each dataset consisted of thirty sequences that were evolved along a phylogeny reconstructed from the 738 non-ambiguous sites of a 30-OTU ingroup subset of the 100-OTU Graphidaceae dataset (the same subset as used below in the comparison of ambiguous region coding methods). GTR+Gamma model parameters were estimated from the real sequence data and used to generate the simulated datasets. The sequences were divided into five partitions. Partition 1 and 2 contained no indels and were each 400 residues long; partition 2 had a substitution rate twice that of partition 1. Partitions 3, 4, and 5 contained indels at a rate of 1 insertion and 2 deletions per 20 substitutions. Indel lengths were randomly generated from a power-law distribution with a slope of 1.6 and a maximum length of 30 residues. The root sequences of partitions 3 and 4 had a length of 100 nucleotides, with partition 3 having a substitution rate twice that of partition 1 and partition 4 thrice that of partition 1; the root sequences of partition 5 had a root length of 200 nucleotides, maximum indel length of 50 residues, and substitution rate twice that of section 1. The resulting partitions 1-2 were unambiguously alignable, whereas partitions 3-5 simulated ambiguous regions of increased length variation and complexity.

### Computing, ordinating, and coding distance and cost score matrices (PICS-Ord)

Ambiguous regions (biological datasets) and partitions containing indels (simulated datasets) were subjected to pairwise alignment to derive distance and cost score matrices. The alignment algorithms implemented in ClustalW2 [48,65], through the web server at the European Bioinformatics Institute (EBI; http://www.ebi.ac.uk/Tools/clustalw2/index.html), were used to derive pairwise sequence identities, as the percentage of non-gapped sites matching in both sequences. To derive distances from the identity scores, the latter were divided by 100 and subtracted from 1. INAASE 3.0 [37] was employed to calculate pairwise cost scores under a given transition:transversion:gap ratio. INAASE encodes each distinct sequence pattern (state) using a single-digit number or letter code and computes a multidimensional step-matrix with the distances between each state. This has the limitation that only up to 32 (64) states can be handled by phylogenetic analysis programs such as PAUP on a 32-bit (64-bit) computer. As an alternative, we employed ARC [33], which is a non-pairwise alignment method based on frequencies of bases and base patterns in each individual sequence.

In addition to simple sequence identity and cost matrices, Ngila 1.3 was applied to find the most likely alignment between two homologous sequences and its log-likelihood score [25,49,66]. Ngila finds optimal alignments between pairs of sequences using a substitution cost matrix and log-affine gap costs (*C_k _*= *a *+ *bk *+ *c*log(*k*)). These costs can be specified directly by the user (the 'cost' model), producing distance scores corresponding to those of INAASE if the same cost scheme is used, or can be derived from an evolutionary model (the 'zeta' model). The zeta model uses a Kimura-2-parameter model [67] to calculate substitution costs and a zeta power-law model to compute log-affine gap costs, which have been shown to be superior to other models of indel evolution [25,49]. We tested both the default options and the free-end-gaps option under Ngila to derive the pairwise distance matrices, and used the latter for the analyses presented here. The free-end-gaps option allows gaps at the start and end of the alignment to have lower or no cost compared to other gaps. This is useful when one expects that the end points of a sequence pair are not necessarily homologous, assumed to be the case in the highly length-variable regions of the ribosomal DNA, especially the ITS1 and ITS2 portions (corresponding to ambiguous regions 1 and 2 in the Physciaceae and Parmeliaceae datasets), which are excised and degraded during the transcription process.

Distance and cost score matrices derived via ClustalW and Ngila were subjected to principal coordinates analysis (PCoA). PCoA is found as a stand-alone application in the freely available executables PCO.exe [[68]; http://www.stat.auckland.ac.nz/~mja/Programs.htm] and DistPCoA.exe [[69]; http://www.esapubs.org/archive/mono/M069/001] and in packages such as R 2.9.2 [[70]; http://www.r-project.org] and the commercial XLSTAT-Pro 2009 http://www.xlstat.com. Since PCoA is an eigenvector analysis, it offers correction for negative eigenvalues if a distance matrix is not metric. Distance matrices derived from sequence identities and cost scores fall under this category. However, the correction is not mandatory, as axes with negative eigenvalues occur at higher orders and hence can be omitted. Our initial tests including correction for negative eigenvalues showed that correction resulted in undesired distortion of the original data, since the number of axes with non-zero eigenvalues will be higher than the number of different sequence patterns in the ambiguous portion. We therefore employed PCoA without correction and retained axes with positive eigenvalues only.

Since fractional ordination scores cannot be used in phylogenetic analyses, we encoded the ordination scores obtained from axes with positive eigenvalues as integers. For each axis, the maximum and mini-mum score (S_max_, S_min_) and the range (S_Range _= S_max _- S_min_) were computed. The maximum range S_Range(max) _across all axes was retained; usually it corresponded to the first axis, more rarely to axes of higher order (because axis variance is determined by both range and dispersion). For each individual OTU, its axis coordinates S_OTU _were then rescaled using the following equation: S_rescaled _= (S_OTU _- S_min_)/S_Range(max)_, which transformed all original scores into values ranging between 0.00 and 1.00. Integer scores INT_OTU _were subsequently computed by multiplying S_rescaled _with 9.99, subtracting 0.495, and rounding to the closest integer value, resulting in 10-state ordered integer scores ranging from 0 to 9. The rescaling by multiplication with 9.99 and subtraction of 0.495 ensures that each integer code represents a nearly equal range of 1.0 prior to rounding. We also explored other scoring schemes by comparing uncoded DNA with recoded data, including 4-state ordered integer scores and 20-state unordered integer scores, and found that 10-state ordered integer scores performed best in terms of preserving phylogenetic signal contained in uncoded DNA.

For the 100-OTU Graphidaceae dataset, we used a simple approach to assess the level of congruence and potential homoplasy between each of the ambiguous regions and the non-ambiguous alignment portion. For all 100 OTUs, Clustal pairwise sequence identity scores were computed for each ambiguous region of the alignment and for the non-ambiguous portion. The resulting distance matrices were plotted against each other and the degree of linear correlation was assessed by means of the Pearson product-moment correlation coefficient as implemented in STATISTICA 6.0.

### Comparative analysis of coding methods using a non-ambiguously aligned biological dataset

To compare the output of coding methods with original, non-coded DNA data, we used a subset of 31 OTUs of the Graphidaceae dataset and the non-ambiguous portion of the alignment, trimmed to 720 positions. The number of 31 OTUs (30 ingroup plus one outgroup) was chosen to accommodate the limitations of INAASE, which can only handle up to 32 distinct sequences patterns per alignment portion. The alignment was divided into 12 portions of 60 positions each, and each portion was subjected to recoding using: (1) INAASE cost scores (step matrix) with a transition: transversion:gap ratio of 1:1:1; (2) ARC; (3) PICS-Ord with Clustal pairwise identity scores (default ratio of 1:1:1); and (4) PICS-Ord with Ngila pairwise log likelihood cost scores (zeta power-law model with default settings); the latter two ordinated with uncorrected PCoA retaining axes with positive eigenvalues only and rescaled as ordered 10-state integer codes. The encoded datasets resulted in 12 characters (step matrices) for INAASE, 276 for ARC, 204 for PICS-Ord with Clustal scores, and 141 for PICS-Ord with Ngila scores, as compared to 364 parsimony informative sites in the original DNA matrix. The original DNA alignment and all encoded datasets were subjected to maximum parsimony analysis in PAUP* 4.0b10 [39], using a heuristic search with tree bisection-reconnection (TBR) branch swapping, the MulTrees option in effect, and 100 random addition sequence replicates. Encoded characters were treated as ordered under PICS-Ord. Bootstrapping was performed with 100 bootstrap and 100 random addition sequence replicates. Tree drawing was carried out in MESQUITE 2.7 [71]. Trees were compared using the relative Robinson-Foulds distance [72,73] as well as clade support values and the presence/absence of specific clades.

### Comparative analysis of PICS-Ord coding versus ambiguous regions excluded or automatically aligned

Using the 100-OTU Graphidaceae dataset, the 705-OTU Physciaceae dataset, and the simulated datasets, we performed a comparative analysis of the multiple alignments as follows: (1) ambiguous regions excluded, and (2) ambiguous regions encoded using PICS-Ord. For option (1), we used the non-ambiguous portions of the two biological datasets and the non-ambiguous partitions 1-2 of the simulated datasets. For option (2), ambiguous regions (biological datasets) or partitions (simulated data) were pairwise aligned and Ngila log likelihood cost scores were computed under the zeta power-law model (default settings). The cost score matrices were ordinated using PCoA without correcting for non-metricity and all axes with positive eigenvalues were retained. Ordination scores were rescaled to 10-state ordered integer codes.

The biological datasets were analyzed under maximum likelihood using the most recent version 7.2.6 of RAxML [[73]; http://wwwkramer.in.tum.de/exelixis/software.html]. This version allows for the combined analysis of DNA and non-DNA characters, the latter under a general MK or GTR model or as ORDERED. The DNA datasets from options (1) and (2) were analyzed under a GTR+G model. The mixed datasets from option (3) were partitioned and the DNA partition was analyzed under a GTR+G model, whereas the encoded partition was subjected to the GTR model with equal rates and fixed (equal) character state frequencies. Rapid bootstrapping was performed under GTR-CAT with 100 replicates.

Phylogenetic inferences on simulated datasets were conducted using the SSE3-vectorized version of RAxML 7.2.6 [74]. On each dataset we conducted 20 ML searches on randomized stepwise addition parsimony trees under the GAMMA model of rate heterogeneity to obtain the best-known ML tree; ML optimization is NP-hard [75]. Datasets were analyzed as follows: (a) partitions 1-4 or 1-2+5 pre-aligned as simulated (unchanged), (b) partitions 1-2 pre-aligned as simulated and 3-5 excluded (corresponding to option 1 above), (c) partitions 1-2 pre-aligned as simulated and 3-4 or 5 encoded using PICS-Ord with Ngila log likelihood cost scores under the zeta power-law model (default settings; corresponding to option 3 above). For the PICS-Ord-encoded partitions of the alignment, we compared GTR, MK, and ORDERED multi-state models as implemented in RAxML 7.2.6. All characters were treated as unweighted. We also tested joint and per-partition branch length estimates for the alignment partitions and found that inferences using joint branch length estimates across all partitions yielded slightly more accurate trees. Conducting 20 ML tree searches with RAxML under the most complex model with respect to computational complexity (PICS-Ord multi-state partitions analyzed under a GTR model) took on average eight minutes on a single AMD Shanghai core running at 2.7 GHz.

To compute the topological distances of all resulting trees to the true tree, we used the respective RAxML option (-f r) to obtain the relative Robinson-Foulds (RF) distance [72,73]. We used Phyutility [76] for some of the batch data manipulations, including batch file format conversions. Tree drawing was carried out in MESQUITE 2.7 [71].

### PICS-Ord Implementation

A reference implementation of PICS-Ord is available from http://scit.us/projects/ngila/wiki/PICS-Ord/. It requires the statistical software package, R (R Development Core Team, 2009), and the alignment software, Ngila [49]. It is flexible but defaults to using Ngila's zeta cost scores to construct distance matrices, and R's cmdscale function to ordinate these matrices. It supports nucleic-acid and protein data and can produce encodings using up to 64 characters. It integrates well with RAxML [73].

## Authors' contributions

RL provided the idea of the novel methodology presented here and performed initial computations under parsimony, as well as the individual Ngila and PCoA analyses and ordination scores recoding and ML analyses of the 706-OTU and 1814-OTU biological datasets. BPH and RL further developed the idea and made analytical comparisons with alternative ambiguous region coding methods. AS developed an updated version of RAxML (7.2.6 and higher) to allow for phylogenetic analysis of ambiguous region codes using a mixed model under maximum likelihood, and performed ML analyses, including computation of RF values, on the simulated datasets and part of the biological datasets. RAC developed an updated version of Ngila for pairwise alignment score matrices to be directly analyzed by PCoA, generated the simulated datasets, and wrote an R script for automated recoding using the PICS-Ord approach. All authors contributed equally to writing the manuscript and read and approved the final manuscript.
